# ARMC Subfamily: Structures, Functions, Evolutions, Interactions, and Diseases

**DOI:** 10.3389/fmolb.2021.791597

**Published:** 2021-11-29

**Authors:** Yutao Huang, Zijian Jiang, Xiangyu Gao, Peng Luo, Xiaofan Jiang

**Affiliations:** ^1^ Department of Neurosurgery, Xijing Hospital, Fourth Military Medical University, Xi’an, China; ^2^ Institue of Neurosurgery of People’s Liberation Army of China (PLA), PLA’s Key Laboratory of Critical Care Medicine, Xijing Hospital, Fourth Military Medical University, Xi’an, China; ^3^ Department of Hepato-biliary Surgery, Xijing Hospital, Fourth Military Medical University, Xi’an, China; ^4^ State Key Laboratory of Cancer Biology, Fourth Military Medical University, Xi’an, China

**Keywords:** Armadillo repeat-containing protein, ARMC, structure, biological function, evolution, interaction, disease, ACVMPs

## Abstract

Armadillo repeat-containing proteins (ARMCs) are widely distributed in eukaryotes and have important influences on cell adhesion, signal transduction, mitochondrial function regulation, tumorigenesis, and other processes. These proteins share a similar domain consisting of tandem repeats approximately 42 amino acids in length, and this domain constitutes a substantial platform for the binding between ARMCs and other proteins. An ARMC subfamily, including ARMC1∼10, ARMC12, and ARMCX1∼6, has received increasing attention. These proteins may have many terminal regions and play a critical role in various diseases. On the one hand, based on their similar central domain of tandem repeats, this ARMC subfamily may function similarly to other ARMCs. On the other hand, the unique domains on their terminals may cause these proteins to have different functions. Here, we focus on the ARMC subfamily (ARMC1∼10, ARMC12, and ARMCX1∼6), which is relatively conserved in vertebrates and highly conserved in mammals, particularly primates. We review the structures, biological functions, evolutions, interactions, and related diseases of the ARMC subfamily, which involve more than 30 diseases and 40 bypasses, including interactions and relationships between more than 100 proteins and signaling molecules. We look forward to obtaining a clearer understanding of the ARMC subfamily to facilitate further in-depth research and treatment of related diseases.

## 1. Introduction

In 1989, an Armadillo repeat-containing protein (ARMC) was first discovered in the polar gene fragment of *Drosophila* (Riggleman et al., 1989), and since then, an increasing number of ARMCs, including the well-known β-catenin, plakoglobin, and plakophilin, have been reported and studied. A growing number of studies have indicated that ARMCs, such as Yelo13p (Vac8p) in yeast (Schneiter et al., 2000), ARC1 in Brassicaceae (Zhang et al., 2014), AtPUB14 in Arabidopsis (Andersen et al., 2004), and Gudu in *Drosophila* (Cheng et al., 2013), are widely distributed in eukaryotes, ranging from fungi and plants to animals. ARMCs comprise a large family of proteins with tandem repeats with a length of approximately 42 amino acids. In general, tandem repeats of amino acids form a substantial binding platform for many proteins that allow degradation of the target protein (e.g., α-catenin (Suzuki et al., 2008)) or activation of the target protein (e.g., the MFF/FIS1 complex and some transcriptional activators (Chen et al., 2019)). The universality and conservation of ARMCs provide convenience and practical feedback for research. Some researchers have studied ARMCs in organisms such as *Drosophila*, TgARO, and *Xenopus* to deduce the similar function of ARMCs in humans and thus attempt to treat related diseases.

The ARMC subfamily consists of ARMC1∼10, ARMC12, and ARMCX1∼6, which are relatively conserved in vertebrates and highly conserved in mammals, particularly primates. Humans harbor the ARMC1∼10 and ARMC12 genes, which encode the ARMC1∼10 and ARMC12 proteins, respectively. The ARMC11 gene is expressed in mice and encodes Maestro heat-like repeat family member 9. The ARMCX cluster genes (ARMCX1∼6), which are highly expressed in the developing and adult nervous systems (López-Doménech et al., 2012) and are unique to mammals, evolved from the retroposition of a single ancestor gene, ARMC10, and may have many similar functions to ARMC1∼12. Recently, an increasing number of researchers have focused on the ARMC subfamily, and a growing number of studies have noted that the ARMC subfamily plays an important role in cell adhesion, intracellular signal transduction, cytoskeleton regulation, mitochondrial function regulation, ciliary movement regulation, embryonic development, and tumor development, among other processes. It has been more than 20 years since ARMCs were first proposed and classified by the German scientist Mechthild Hatzfeld in 1998 (Hatzfeld, 1999). Moreover, it has been 10 years since Tewari et al. (2010) further supplemented the newly identified ARMCs and extended the distribution of ARMCs to unicellular eukaryotes. The updating of ARMCs is urgently needed. Here, we focus on the ARMC subfamily (ARMC1∼10, ARMC12, and ARMCX1∼6), which is relatively conserved in vertebrates and highly conserved in mammals, particularly primates (ACVMPs). We review the structures, biological functions, evolutions, interactions and related diseases of this ARMC subfamily, which involve more than 30 diseases and 40 bypasses, including interactions and relationships between more than 100 proteins and signaling molecules (Table 1).

**TABLE 1:** ARMC subfamily, diseases, and pathways.

Name	Disease	Pathway
ARMC1	AD^*^, PD^*^, T2B^*^, cancer^*^, etc.	ARMC1/SLC25A46^*^
ARMC2	COPD	NRF1/ARMC2/PYCARD/caspase&NF-κB^*^
ARMC3	HT	AMRC3/CRACR2A/ORAI1&STIM1^*^
	PCD	FOXJ1/ARMC3&CFAP157
	infertility	ARMC3/PKIA/PKA/AKAP3^*^
ARMC4	CC, NMD, cancer	ARMC4/GSK3B/Wnt/β-catenin^*^
	PCD, CD	CCDC151/ARMC4/ODA
	ALS, AD^*^, PD^*^	ARMC4/GSK3B/Drp1^*^
		ARMC4/PKA/Wnt/β-catenin^*^
ARMC5	PBMAH, PA, cancer	CUL3/ARMC5/cyclin E
		ARMC5/PKA/Wnt/β-catenin/Axin2&Lef1&Cyclin D1
ARMC6	NB	MYCN&MAX/ARMC6/NOTCH/NICD/CSL^*^
ARMC7	AD, CAA^*^	ARMC7/APP/clusterin/p53/Dkk1/Wnt/PCP/JNK/EGR1^*^
ARMC8	Cancer	ARMC8/TGF-β/Wnt/β-catenin/cyclin D1&MMP7&c-Myc
		ARMC8/α-catenin/E-cadherin
ARMC9	JS, PCD	TOGARAM1/ARMC9
	T2B^*^	ARMC9/FBP^*^
	MF^*^, OI^*^, cancer^*^	ARMC9/DCR2/CLN3&SWI4^*^
ARMC10	Cancer	ARMC10/EPHA1/ILK/RHOA/ROCK/VEGF&MMP^*^
		ARMC10/TCEA2&SMARCD1/Wnt/β-catenin^*^
	AD^*^, PD^*^, T2B^*^, cancer^*^, CAA^*^	ARMC10/KIF5/Miro1-2/Trak2/Aβ
		AMPK/ARMC10/MFF&FIS1&Drp1
ARMC12	NB	ARMC12/RBBP4/PRC2/EZH2/CADM1&EGLN3&HRK&HS6ST3&SMAD9
ARMCX1	Cancer	Wnt/β-catenin/CREB/ARMCX1/Bax&caspase&Bcl-2
		ARMCX1/PAR-1/ρGTPase
		ARMCX1/JAK1/STAT3
	Leukemia^*^, MDS^*^, cancer^*^, AID^*^	ARMCX1/EZH2^*^
ARMCX2	FXS	ARMCX2/EZH2/FMR1^*^
	Cancer	ARMCX2/Wnt/NF-κB
ARMCX3	Cancer	Wnt/PKC/ARMCX3
		ARMCX3/Wnt/β-catenin/TCF/LEF
		ARMCX3/AKT/Slug/E-cadherin
	NA^*^, depression^*^, HAD^*^, cancer^*^, AD^*^	ARMCX3/Sox10/nAChR
	AD^*^, PD^*^, T2B^*^, cancer^*^, etc.	ARMCX3/KIF5/Miro/Trak2
	WS^*^, GBS^*^	ARMCX3/SOX10/DUSP15&MYRF^*^
	T2B^*^	ARMCX3/HDAC7&FYN
ARMCX4	Cancer, CMS^*^, GD^*^, infertility, NMD^*^	ARMCX4/DPAG1^*^
ARMCX5	XDS	
ARMCX6	Cancer	

The superscript ^*^ indicates predicted diseases or pathways.

AD, Alzheimer’s disease; AID, autoimmune disease; ALS, amyotrophic lateral sclerosis; CAA, cerebral amyloid angiopathy; CC, cryptorchidism; CD, ciliary dyskinesia; CMS, congenital myasthenic syndrome; COPD, chronic obstructive pulmonary disease; FXS, fragile X syndrome; GBS, Guillain-Barre syndrome; GD, glycosylation disease; HAD, hyperactivity disorder; HT, hypertension; JS, Joubert syndrome; MF, malformation; MDS, myelodysplastic syndrome; NA, nicotine addiction; NB, neuroblastoma; NMD, neonatal maxillofacial deformity; OI, osteogenesis imperfecta; PA, primary aldosteronism; PBMAH, primary bilateral macronodular adrenal hyperplasia; PCD, primary ciliary dyskinesia; PD, Parkinson’s disease; T2B, type 2 diabetes; WS, Waardenburg syndrome; XDS, Xq22.1 deletion syndrome.

## 2. Characteristic Structures, Functions, and Evolution of the ARMC Subfamily

### 2.1. Basic Structure of the ARMC Subfamily

**FIGURE 1:**
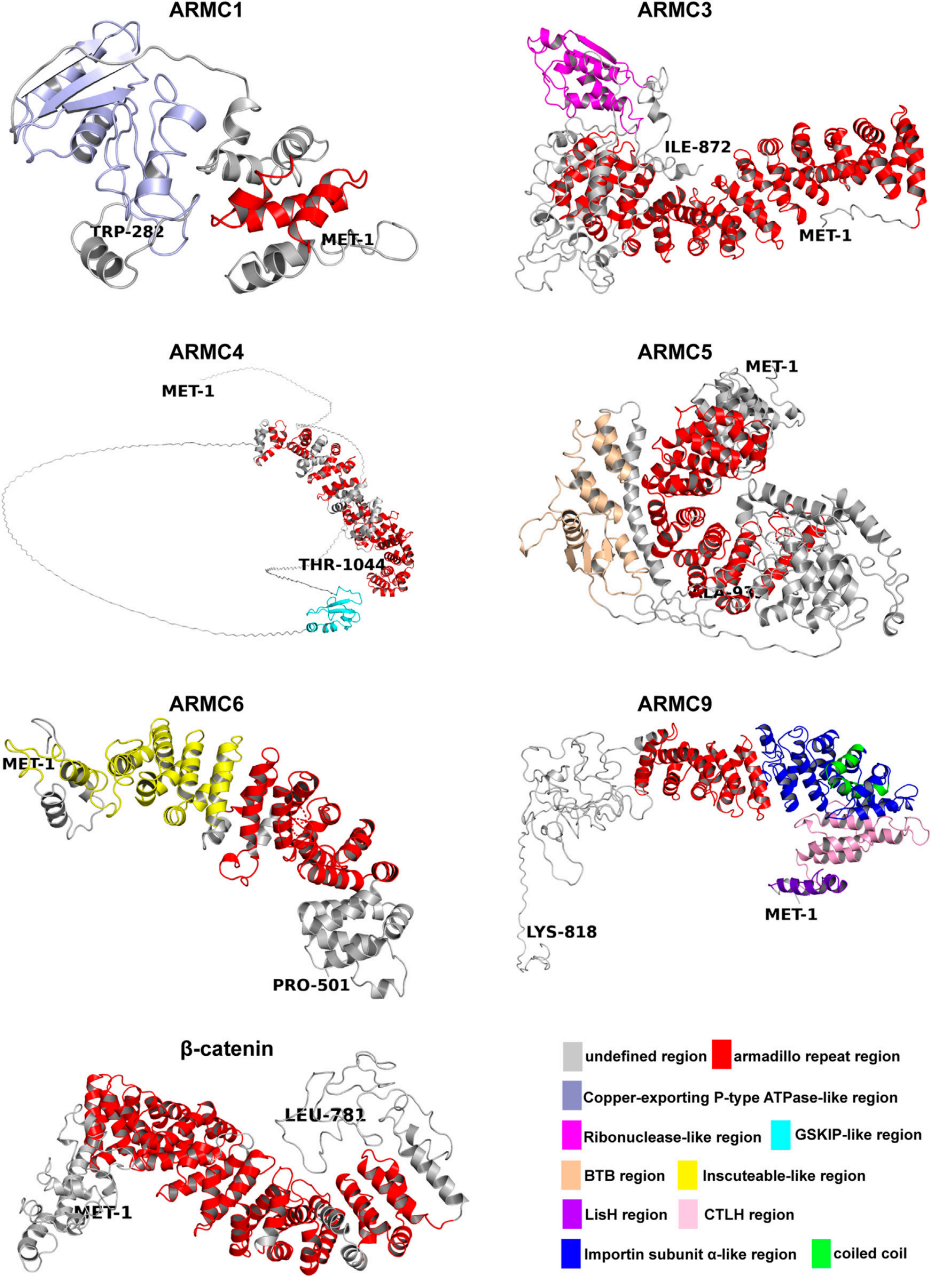
Structures of the ARMC subfamily. The N-termini are shown as MET-1, and the C-termini are shown as another amino acid. Depicted are the undefined region (gray), Armadillo repeat region (red), Copper-exporting P-type ATPase-like region (light blue), Ribonuclease-like region (magenta), GSKIP-like region (cyan), BTB region (wheat), Inscuteable-like region (yellow), LisH region (purple), CTLH region (pink), Importin subunit α-like region (blue), and coiled coil region (green). All structures (homology models) were generated using Phyre2 (Kelley et al., 2015). The pictures were generated using PyMOL (Schrodingerhe).

The canonical ARMC (e.g., β-catenin) contains a central armadillo repeat, and the noncanonical ARMC (e.g., ARMC1) contains special N-terminal or C-terminal domains in addition to the central armadillo repeat. β-catenin, as a canonical ARMC, is almost entirely occupied by its armadillo repeat domain, whereas ACVMPs, as noncanonical ARMCs, are partly occupied by an armadillo repeat domain with additional domains on the N- or C-terminus (Figure 1). Regardless of the number of armadillo repeat sequences, the armadillo repeat region, sometimes in combination with adjacent sequences (e.g., in ARMC1 and ARMC6), forms an α-superhelix (Figure 1). For some ACVMPs, such as ARMC1 and ARMC6, which have few armadillo repeat sequences, this structure can greatly expand the functional region of the armadillo repeat to prepare the binding platform by forming an α-superhelix along with adjacent sequences.

**FIGURE 2:**
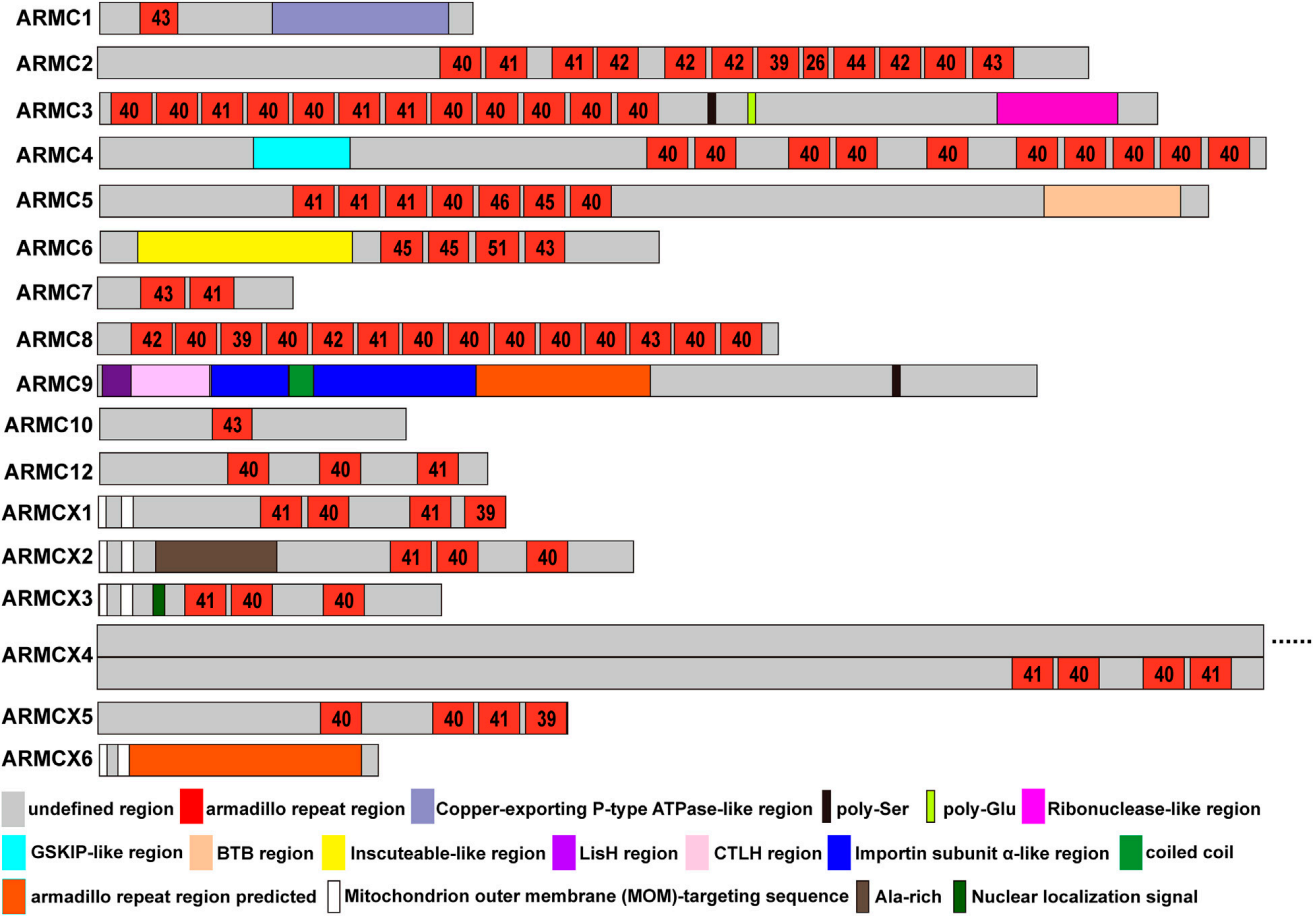
ARMC subfamily. The length and location of each domain in the ARMC subfamily are roughly depicted. Shown are the undefined region (gray), Armadillo repeat region (red), Copper-exporting P-type ATPase-like region (light blue), poly-Ser (black), poly-Glu (limon), Ribonuclease-like region (magenta), GSKIP-like region (cyan), BTB region (wheat), Inscuteable-like region (yellow), LisH region (purple), CTLH region (pink), Importin subunit α-like region (blue), coiled coil region (green), predicted Armadillo repeat domain (orange), MOM-targeting sequence (white), Ala-rich region (sand), and nuclear localization signal (forest). The numbers in the checks represent the number of amino acids in the armadillo repeat domain.

Many ACVMPs have additional domains on the N- or C-terminus that canonical ARMCs usually do not contain (Figures 1, 2). For example, the C-terminus of ARMC1 is similar to that of the copper-exporting P-type ATPase (PDB entry: 2RML): three β-sheets with two α-helices on the opposite side form a cylinder-like tube with an empty center for binding (Figure 1). The C-terminus of ARMC3 is analogous to that of ribonuclease (Yee et al., 1994) (PDB entry: 4XZ7): several α-helixes are tangled across with few β-sheets on the opposite side (Figure 1). The N-terminus of ARMC4, which is similar to GSK3B-interacting protein (GSKIP, PDB entry: 1SGO), consists of three to four β-sheets along with an α-helix that folds at a right angle on its side. The special domain on the C-terminus of ARMC5 is similar to that of speckle-type POZ protein (PDB entry: 3HU6), which contains a Brac, Tramtrack, Broad-complex (BTB) domain, three β-sheets and a string of α-helixes; among these α-helixes, three α-helixes surround the β-sheets, and four α-helixes besiege a virtual tube to form a hollow cuboid-like structure (Figure 1). The additional domain on the N-terminus of ARMC6 is analogous to that of Inscuteable (INSC, PDB entry: 5A7D) and contains a string of α-superhelices (Figure 1). The N-terminus of ARMC9 has two different functional domains: a structure similar to glucose-induced degradation protein 8 (GID8, PDB entry: 6SWY) contains two parts, a LisH domain and a CTLH domain (Figures 1, 2), and a structure analogous to Importin subunit alpha (PDB entry: 1WA5), which consists of an α-superhelix (Figures 1, 2). The center of the α-superhelix contains a coiled coil.

ACVMPs, which consist of ARMC1∼10, ARMC12, and ARMCX1∼6, contain a central domain of tandem repeats with a length of approximately 42 amino acids (Figure 2). However, there are some exceptions; for instance, ARMC2 has a repeat with a length of 26 amino acids at the eighth repeat that is markedly shorter than that found in other ARMCs, and ARMC6 has a repeat of 51 amino acids in length at the third repeat that is markedly longer than that in any other ARMC (Figure 2). The ARMCX cluster usually contains two mitochondrial outer membrane (MOM)-targeting sequences in its N-terminus to aid its localization to the outer mitochondrial membrane. Furthermore, ARMCX3 contains a nuclear localization signal that helps ARMCX3 shuttle between the nucleus and cytoplasm. Other sites, such as poly-Ser, poly-Glu, and Ala-rich sites, may be substantial phosphorylation sites.

### 2.2. Structural Function of the ARMC Subfamily

Because ACVMPs have not only an armadillo repeat domain in the center but also additional domains in the C- or N-terminus, ACVMPs may have two basic functions: a canonical protein-binding function based on the armadillo repeat domain and additional functions related to the terminals. For example, the C-terminus of ARMC1 harbors a heavy-metal-associated domain (Figure 1) containing two conserved cysteines that is involved in the binding of metals and is usually found in proteins that transport or detoxify heavy metals (Radford et al., 2003). This finding suggests that ARMC1 binds to a metal ion and participates in metal ion transport or detoxification through its C-terminus. The C-terminus of ARMC3 is analogous to that of ribonuclease (Yee et al., 1994) (Figure 1), which suggests that ARMC3 functions partly as a hydrolase or transferase. On the one hand, the N-terminus of ARMC4, which is similar to GSKIP (Figure 1), can interact with GSK3B to help control the destabilizing phosphorylation of β-catenin and thus inhibit the Wnt/β-catenin pathway. On the other hand, the N-terminus of ARMC4 can directly interact with PKA to help control the stabilizing phosphorylation of β-catenin and thus promote the Wnt/β-catenin pathway (Chou et al., 2006; Dema et al., 2016). The BTB domain on the C-terminus of ARMC5 and on many proteins that harbor the BTB domain, which is important for binding to substrate proteins and homodimerization, is involved in the ubiquitination and proteasomal degradation of BRMS1, DAXX, PDX1/IPF1, GLI2, and GLI3, among other proteins, in complex with CUL3 (Kwon et al., 2006; Kim et al., 2011). This finding suggests that the BTB domain on ARMC5, similar to speckle-type POZ protein, can interact with CUL3 to form the ubiquitin ligase complex E3 and thus mediate the ubiquitination of target proteins, which most often leads to their proteasomal degradation (Furukawa et al., 2003; Kim et al., 2011). The N-terminus of ARMC6, which is similar to Inscuteable, is important for cytoskeleton organization, cell division, and nervous system development and is needed for protein localization, spindle orientation, and RNA localization (Kraut and Campos-Ortega, 1996; Li et al., 1997; Cai et al., 2003). The N-terminus of ARMC9 has more than one additional domain. When the concentration of glucose increases, the first domain, a domain similar to one in GID8, meditates the ubiquitin-proteasome-dependent catabolite degradation of fructose-1,6-bisphosphatase (FBP) and thus regulates glucose balance (Regelmann et al., 2003). The second domain contains a structure analogous to that of subunit alpha in Importin, a nuclear localization signal (NLS) receptor that binds specifically and directly to substrates containing either a simple or bipartite NLS motif and promotes the docking of import substrates to the nuclear envelope (Küssel and Frasch, 1995). Furthermore, the GID8-like region of ARMC9 may interact with DCR2 and thus the phosphatase CLN3 or SWI4 to promote cell cycle progression (Pathak et al., 2004). This finding suggests that ARMC9 regulates glycometabolism and the cell cycle and participates in nuclear protein transport via its N-terminal regions.

### 2.3. Functional Evolution of the ARMC Subfamily

#### 2.3.1 ARMC1

ARMC1 is highly conserved in mammals. For example, although the Intro sequences and UTR of the ARMC1 gene in gorilla are different from those in *Homo sapiens*, the exon sequences are the same. Therefore, the ARMC1 protein of gorilla is the same as that of *Homo sapiens*. Similarly, the ARMC1 protein of *Mus musculus* exhibits 98% identity with that of *Homo sapiens*, and only 5 amino acids are different. This conservation suggests that ARMC1 may play a common and similar role in mammals. In fact, it has been reported that ARMC1 is a subunit of the mammalian mitochondrial contact site and cristae organizing system/mitochondrial intermembrane space bridging complex (MICOS/MIB) (Wagner et al., 2019). This function may rely on the armadillo repeat domain and the Copper-exporting P-type ATPase-like region on the C-terminus (Figures 1, 2). In vertebrates (nonmammals), such as zebrafish, the situation is serious. The ARMC1 protein of zebrafish exhibits 79% identity with that of *Homo sapiens*, and several amino acids are different, particularly those in the armadillo repeat domain and the Copper-exporting P-type ATPase-like region on the C-terminus. It suggests that the mitochondrial function of zebrafish may be different from those of *Homo sapiens*, gorilla, and *Mus musculus*.

#### 2.3.2 ARMC2

ARMC2 is highly conserved in primates. The ARMC2 protein of gorilla exhibits 99% identity with that of *Homo sapiens*, whereas ARMC2 proteins in mammals (nonprimates), such as *Mus musculus*, show 73% identity with that of *Homo sapiens*. The ARMC2 protein of vertebrates (nonmammals), such as zebrafish, exhibits an identity of only 43% with that of *Homo sapiens*. This finding suggests that ARMC2 evolved gradually from nonmammals to mammals and primates and may play a special role in primates. A genome-wide association and large-scale follow-up study revealed an association for ARMC2 with pulmonary function and chronic obstructive pulmonary disease (COPD) (Soler Artigas et al., 2011). Zebrafish have no lungs, whereas the pulmonary function of *Mus musculus* is weaker than that of *Homo sapiens* and gorilla due to size and exercise. This finding may partly explain, from the perspective of biological function, the difference in ARMC2 proteins among primates, mammals, and vertebrates.

#### 2.3.3 ARMC3

ARMC3 is highly conserved in primates. The ARMC3 protein of gorilla exhibits 99% identity with that of *Homo sapiens*, whereas the ARMC3 proteins in mammals (nonprimates), such as *Mus musculus*, exhibits 81% identity with that of *Homo sapiens*. The ARMC3 protein of vertebrates (nonmammals), such as zebrafish, exhibits an identity of only 49% with that of *Homo sapiens*. This finding suggests that ARMC3 evolved gradually from nonmammals to mammals and primates and may play a special role in primates. The ARMC3 gene of *Homo sapiens* and gorilla has 19 exons. However, 20 exons are found in the ARMC3 gene of *Mus musculus*, and the additional exon is inserted between the 16th and 17th exons. As a result, an additional structure is found on the C-terminus of ARMC3 between poly-Ser and poly-Glu (Figure 2). Interestingly, the opposite findings have been found in zebrafish. Seventeen exons are found in the ARMC3 gene of zebrafish, and 1st and 16th exons are deleted in this gene. The first exon is the UTR and is not translated into protein. Therefore, this deletion would result in absence of the structure between poly-Ser and poly-Glu at the C-terminus of ARMC3 in zebrafish (Figure 2). This finding suggests that the structure between poly-Ser and poly-Glu may play a special role in evolution. Moreover, it has been reported that the expression of ARMC3 is significantly increased in highly active sperm (D’Amours et al., 2019), whereas the deletion of exon 11 of the ARMC3 gene may alter the reading frame, leading to premature termination of translation, which causes sperm defects and thus male infertility (Pausch et al., 2016). Interestingly, ARMC3 exon 11 encodes amino acids 476–521 of ARMC3, which are located across the end of the last armadillo repeat sequence (Figures 1, 2). Therefore, the deletion of exon 11 in ARMC3 will lead to absence of the sequence after the last armadillo repeat in the C-terminal region, which is analogous to that in ribonuclease (Figures 1, 2).

#### 2.3.4. ARMC4

ARMC4 is highly conserved in primates. The ARMC4 protein of gorilla exhibits 98% identity with that of *Homo sapiens*, whereas the ARMC4 proteins in mammals (nonprimates), such as *Mus musculus*, exhibits 85% identity with that of *Homo sapiens*. The ARMC4 proteins in vertebrates (nonmammals), such as zebrafish, exhibit an identity of only 58% with that of *Homo sapiens*. Moreover, although the ARMC4 protein of *Mus musculus* exhibits only 85% similarity to that of *Homo sapiens*, the armadillo repeat domains of ARMC4 of *Mus musculus* exhibits 99% similarity to those of ARMC4 of *Homo sapiens*. Similarly, although the ARMC4 protein of zebrafish exhibits only 58% identify with that of *Homo sapiens*, the armadillo repeat domains of ARMC4 of zebrafish exhibit 75% similarity to those of *Homo sapiens* ARMC4, whereas the non-armadillo repeat domains of zebrafish ARMC4 are almost completely different from those of *Homo sapiens* ARMC4. This finding suggests that although ARMC4 evolved gradually from nonmammals to mammals and primates, its armadillo repeat domains are relatively conserved. In fact, ARMC4 is an axon protein that is necessary for the correct positioning and anchoring of outer dynein arms (ODAs) (Hjeij et al., 2013). Therefore, the armadillo repeat domains of ARMC4 may play an important role in the correct positioning and anchoring of ODAs in various species, ranging from nonmammals to mammals and primates.

#### 2.3.5. ARMC5

ARMC5 is highly conserved in primates. The ARMC5 protein of gorilla exhibits 99% identity with that of *Homo sapiens*, whereas ARMC5 proteins in mammals (nonprimates), such as *Mus musculus*, shows 87% identity with that of *Homo sapiens*. The ARMC5 proteins in vertebrates (nonmammals), such as zebrafish, shows an identity of only approximately 30% with that of *Homo sapiens*. Moreover, although the ARMC5 protein of *Mus musculus* exhibits only 87% identify to that of *Homo sapiens*, the armadillo repeat domains and the BTB region of *Mus musculus* ARMC5 exhibit 99% identify to those of *Homo sapiens* ARMC5. This finding suggests that armadillo repeat domains and the BTB region are conserved during the process of evolution from *Mus musculus* to gorilla and *Homo sapiens*, and these domains may play a special role in these mammals. Notably, although the ARMC5 protein of gorilla shows 99% identity to that of *Homo sapiens*, it has an additional sequence of approximately 95 amino acids in the head of the N-terminus because the ARMC5 gene of *Homo sapiens* has six exons, whereas that of gorilla has nine exons. Two additional exons lie in the head of ARMC5, whereas the fourth exon is divided into two exons by an Intron. Interestingly, increases in the species level are associated with increases in ARMC5 protein length. The ARMC5 of *Homo sapiens* has a length of 935 amino acids, whereas the lengths of gorilla and zebrafish ARMC5 proteins are 1,030 and 1,218 amino acids, respectively. This finding suggests that the ARMC5 gene lost some auxiliary or unnecessary functions during the process of evolution, whereas the armadillo repeat domain and BTB region, which are conserved during the process of evolution, may play a common role. In fact, it has been reported that on the one hand, ARMC5 inhibits PKA activation to restrain the Wnt/β-catenin pathway and thus inhibits Axins, Lef1, and Cyclin D1 expression to suppress the cell cycle (Berthon et al., 2017a); on the other hand, the BTB domain in ARMC5 can interact with CUL3, which causes ARMC5 to be ubiquitinated and further degraded by proteases and thereby promotes the cell cycle (Cavalcante et al., 2020). This finding suggests that ARMC5 could regulate the cell cycle positively and negatively, which may explain why the armadillo repeat domain and BTB region were conserved during the process of evolution because the cell cycle and proliferation are indispensable in any life.

#### 2.3.6. ARMC6

ARMC6 is highly conserved in primates. The ARMC6 protein of gorilla exhibits 99% identity with that of *Homo sapiens*, whereas the ARMC6 proteins in mammals (nonprimates), such as *Mus musculus*, shows 83% identity with that of *Homo sapiens*. The ARMC6 proteins in vertebrates (nonmammals), such as zebrafish, has 61% identity with that of *Homo sapiens*. Notably, the ARMC6 gene of *Homo sapiens* has nine exons, whereas those of *Mus musculus* and zebrafish have eight exons and do not harbor the first exon, which leads to absence of the region before the Inscuteable-like region (Figures 1, 2). The Inscuteable-like region plays a major role in controlling asymmetric cell division and activating the NOTCH signaling pathway, which regulates target gene expression through its hydrolysis fragment NICD or through ICN binding with the transcription factor CSL (Udolph et al., 2009). Deletion of the region before the Inscuteable-like region allows it to be exposed directly at the N-terminus, which may lead to dysfunction of the Inscuteable-like region.

#### 2.3.7. ARMC7

ARMC7 is highly conserved in primates. The ARMC7 protein of gorilla exhibits 98% identity with that of *Homo sapiens*, whereas the ARMC7 proteins in mammals (nonprimates), such as *Mus musculus*, exhibits 84% identity with that of *Homo sapiens*. The ARMC7 proteins in vertebrates (nonmammals), such as zebrafish, shows 62% identity with that of *Homo sapiens*. No additional domain is found on the terminus of ARMC7. Therefore, the functions of ARMC7 may be mediated by its interaction with other proteins.

#### 2.3.8. ARMC8

ARMC8 is highly conserved in vertebrates and not only in mammals. The ARMC8 protein of gorilla is the same as that of *Homo sapiens*, and the ARMC8 proteins of mammals (nonprimates), such as *Mus musculus*, exhibits 99% identify to that of *Homo sapiens*. The ARMC8 proteins of vertebrates (nonmammals), such as zebrafish, has an identity of 86% with that of *Homo sapiens*. Moreover, the ARMC8 proteins of *Homo sapiens*, gorilla, *Mus musculus*, and zebrafish have a sequence length of 673 amino acids. This high conservation, from the perspective of structure, benefits from the armadillo repeat domains that occupy almost the entire sequences of ARMC8 proteins. Simultaneously, this high conservation indicates the critical roles of ARMC8 in vertebrates. On the one hand, ARMC8 is a key component of the C-terminal of the lissencephaly type-1-like homology motif (CTLH) complex, which has E-3 ligase activity and is related to all basic biological processes and functions of the PI3 kinase, Wnt, TGF-β, and NF-κB pathways to regulate proliferation, survival, programmed cell death, cell adhesion, migration and other activities (Huffman et al., 2019). On the other hand, ARMC8 binds to the N-terminal sequence of α-catenin (amino acids 82–148) and promotes its degradation (Suzuki et al., 2008) to regulate cell adhesion. Proliferation, survival, programmed cell death, cell adhesion, and migration are nearly all the basic functions of all vertebrate cells, which may explain why ARMC8 is highly conserved in vertebrates from the perspective of biological function.

#### 2.3.9. ARMC9

ARMC9 is highly conserved in primates. The ARMC9 protein of gorilla has 98% identity with that of *Homo sapiens*, whereas the ARMC9 proteins of mammals (nonprimates), such as *Mus musculus*, exhibits 85% identity with that of *Homo sapiens*. The ARMC9 proteins of vertebrates (nonmammals), such as zebrafish, has an identity of 58% with that of *Homo sapiens*. Moreover, although the ARMC9 protein of *Mus musculus* exhibits only 85% identity to that of *Homo sapiens*, the amino acid sequence from 1 to 660 of *Mus musculus* ARMC9 shows 92% identify to that of *Homo sapiens*. Similarly, although the ARMC9 protein of *Mus musculus* exhibits only 58% identify to that of *Homo sapiens*, the amino acid sequence from 1 to 660 of *Mus musculus* ARMC9 shows 65% identity to that of *Homo sapiens*. This finding suggests that the amino acid sequence before the 660th site may play a relatively conservative role in evolution. The structures before the 660th site are mainly the LisH region, CTLH region, Importin subunit α-like region, and armadillo repeat region (Figures 1, 2). It has been reported that the N-terminus of ARMC9, which is similar to GID8, may interact with DCR2 and the phosphatase CLN3 or SWI4 to regulate cell cycle progression (Pathak et al., 2004). The N-terminus of ARMC9, which is analogous to SRP1, interacts with multiple components of the cell nucleus that are needed for mitosis and regulates the normal function of microtubules and spindle pole bodies as well as nuclear integrity (Küssel and Frasch, 1995). Both domains on the N-terminus of ARMC9 may regulate the cell cycle, although through different pathways, which strongly suggests that ARMC9 is correlated with the cell cycle. This finding may explain why the amino acids before the 660th site are relatively conserved from the perspective of biological function.

#### 2.3.10. ARMC10

**FIGURE 3:**
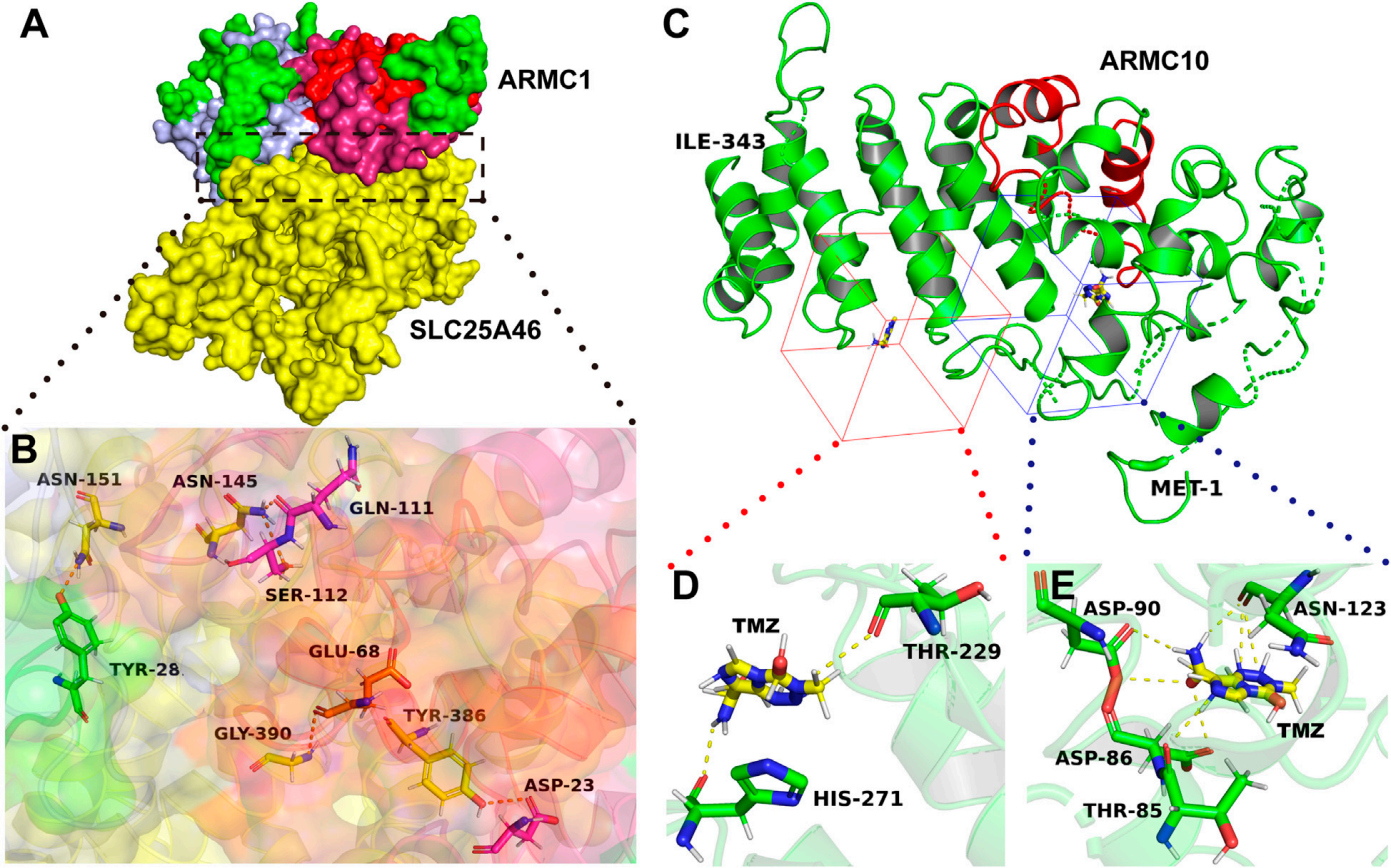
Interaction regions in ARMC proteins. **(A)** ARMC1 interacts with ALS25A46. The yellow color shows SLC25A46, and the other colors shows ARMC1 proteins: the red color shows the armadillo repeat domain of ARMC1, the pink color shows the domain adjacent to the armadillo repeat domain, which combine to form an α-superhelix, and the light blue color shows the heavy metal-associated domain. **(B)** Zoomed-in image. Among the five amino acids in ARMC1 that can form polar bonds with SLC25A46, the armadillo repeat functional region contributes four, ASP-23, GLU-68, GLN-111, and SER-112. The pink sticks show the amino acids in the pink region; the yellow sticks show the amino acid in the yellow region; the orange sticks show the amino acid in the red region; the green stick shows the amino acid in the green region; blue: N atom, red: O atom. **(C)** ARMC10 interacts with TMZ. The N-terminus is shown as MET-1, whereas the C-terminus is shown as another amino acid. The red cartoon represents the armadillo repeat domain. The red and blue cubes show the ligand-binding pockets of ARMC10. **(D,E)** Zoomed-in image. Green: C atom, red: O atom, blue: N-atom. The structures (homology models) of ARMC1, SLC25A46, and ARMC10 were generated using Phyre2 (Kelley et al., 2015), and the structure (crystal model) of TMZ were obtained from The Cambridge Crystallographic Data Centre (CCDC Number: 665060, CSD Entry: DIPGIS11). The pictures were generated using PyMOL (Schrodingerhe LLC) and PlayMolecule (PlayMolecule).

ARMC10 is highly conserved in primates. The ARMC10 protein of gorilla exhibits 99% identity with that of *Homo sapiens*, whereas the ARMC10 proteins of mammals (nonprimates), such as *Mus musculus*, show 70% identity with that of *Homo sapiens*. The ARMC10 proteins of vertebrates (nonmammals), such as zebrafish, have an identity of only 34% with that of *Homo sapiens*. Notably, the ARMC10 protein of *Mus musculus* does not contain the amino acid sequence from the 44th to the 81st site, and the deletion of these amino acid sequence is due to loss of the second exon of the ARMC10 gene of *Mus musculus*. These polar residues are located far before the armadillo repeat domain of ARMC10 and may play a special role in primates. It appears that these polar residues are beneficial for the formation of ligand-binding pockets; for example, THR85, ASP86, ASP90, and ASN123 form noncovalent polar bonds with temozolomide (TMZ) (Figures 3C,E). The amino acid sequence from the 44th to the 81st site, which are next to the ligand-binding pocket, may enhance the stability of the binding, and this enhanced stability results in a deficiency in the concentration of TMZ in patients with glioma who undergo TMZ chemotherapy. This finding may partly explain why ARMC10 leads to TMZ drug-resistant glioma (Cai et al., 2020).

#### 2.3.11. ARMC12

ARMC12 is highly conserved in primates. The ARMC12 protein of gorilla has 99% identity with that of *Homo sapiens*, whereas the ARMC12 proteins of mammals (nonprimates), such as *Mus musculus*, exhibits 84% identity with that of *Homo sapiens*. Vertebrates (nonmammals), for example, zebrafish, do not harbor ARMC12.

#### 2.3.12. ARMCX Cluster

ARMCX1 and ARMCX2 are highly conserved in primates. The ARMCX1 and ARMCX2 proteins of Pongo abelii exhibit identities of 98 and 99%, respectively, with those of *Homo sapiens*, whereas the ARMCX1 and ARMCX2 proteins of mammals (nonprimates), such as *Mus musculus*, have 84 and 78% identities, respectively, with those of *Homo sapiens*. Vertebrates (nonmammals), such as zebrafish, do not harbor ARMCX1 or ARMCX2. Moreover, although the ARMCX1 and ARMCX2 proteins of *Mus musculus* exhibit only 84 and 78% identity, respectively, with those of *Homo sapiens*, the armadillo repeat domains and mitochondrial outer membrane (MOM)-targeting sequences of ARMCX1 and ARMCX2 of *Mus musculus* show 99% identify with those of *Homo sapiens*, respectively. This finding suggests that armadillo repeat domains and MOM-targeting sequences are conserved during the process of evolution from *Mus musculus* to Pongo abelii and *Homo sapiens* and may play a common role in these mammals. In fact, EZH2 is a polycomb group (PcG) protein and a catalytic subunit of the PRC2/EED-EZH2 complex, which methylates “Lys-9” (H3K9me) and “Lys-27” (H3K27me) of histone H3 and thereby leads to transcriptional repression of the affected target gene (McCabe et al., 2012; Hübner et al., 2019). ARMCX1 may interact with EZH2, and this interaction leads to target gene methylation and thus inhibition of the regeneration of hematopoietic stem cells (STRING, 2021; Pathway Commons, 2019; Pierce et al., 2014). The same may also be true for ARMCX2 because the length of the whole sequence and the lengths of the two armadillo repeat domains of ARMCX1 and ARMCX2 are very similar. This finding may partly explain, from the perspective of biological function, why armadillo repeat domains and MOM-targeting sequences are relatively conserved during the process of evolution.

ARMCX3 is highly conserved in mammals and not only in primates. The ARMCX3 protein of Pongo abelii has an identity of 99% to that of *Homo sapiens*, with only one different amino acid, whereas the ARMCX3 proteins of mammals (nonprimates), such as *Mus musculus*, exhibits 97% identity to that of *Homo sapiens*. Vertebrates (nonmammals), such as zebrafish, do not harbor ARMCX3. This finding suggests that armadillo repeat domains and MOM-targeting sequences are conserved in the process of evolution from *Mus musculus* to Pongo abelii and *Homo sapiens* and may play a common role in these mammals. In fact, it has been reported that ARMCX3 interacts with the Kinesin/Miro/Trak2 complex in a calcium-dependent manner to regulate neuronal mitochondrial dynamics and transport (López-Doménech et al., 2012). The Wnt/PKC pathway also indirectly regulates the distribution and dynamics of mitochondria by degrading ARMCX3 (Serrat et al., 2013). Mitochondrial function is nearly the basis of all mammals, which may partly explain why ARMCX3 is highly conserved in mammals.

ARMCX4 is highly conserved in primates. The ARMCX4 protein of gorilla has 98% identity with that of *Homo sapiens*, whereas the ARMCX4 proteins of mammals (nonprimates), such as *Mus musculus*, shows approximately 55% identity with that of *Homo sapiens*. Vertebrates (nonmammals), such as zebrafish, do not harbor ARMCX4. Moreover, although *Mus musculus* ARMCX4 shows only 55% identity to that of *Homo sapiens*, the identity of the armadillo repeat domains of ARMCX4 in *Mus musculus* is 88% to those of *Homo sapiens* ARMCX4. This finding suggests that armadillo repeat domains are conserved in the process of evolution from *Mus musculus* to gorilla and *Homo sapiens* and may play a common role in these mammals. In fact, it has been reported that ARMCX4 promotes the differentiation of spermatogonial stem cells (SSCs), and ARMCX4 mutations can cause male infertility. This finding suggests that the armadillo repeat domain may be critical for the differentiation of SSCs and also partly indicates, from the perspective of biological function, why this domain is relatively conserved in the revolution.

ARMCX5 and ARMCX6 are highly conserved in primates. The ARMCX5 and ARMCX6 proteins of gorilla share 99% identity with those of *Homo sapiens*, and the ARMCX5 and ARMCX6 proteins of mammals (nonprimates), such as *Mus musculus*, exhibits 57 and 70% identity, respectively, with those of *Homo sapiens*. Vertebrates (nonmammals), for example, zebrafish, do not harbor ARMCX5 or ARMCX6. Partial deletion of the ARMCX5 gene may lead to Xq22.1 deletion syndrome and cause epilepsy, cleft palate, intellectual disability, respiratory failure, and female heterozygous development defects (Zhou et al., 2014; Cao and Aypar, 2016).

## 3. Functional Interactions of the ARMC Subfamily in Specific Diseases

### 3.1. Diseases of the Respiratory System

#### 3.1.1. Chronic Obstructive Pulmonary Disease (COPD)

A genome-wide association and large-scale follow-up study revealed an association for ARMC2 with pulmonary function and COPD (Soler Artigas et al., 2011). COPD, a major cause of death and morbidity worldwide, may be caused by unregulated inflammation, autoimmune processes within lung tissue, a proteolysis/antiproteolysis imbalance, destroyed repair mechanisms, and deviated microbiota, among which unregulated inflammation and autoimmune processes within the lung tissue are the most important (Bagdonas et al., 2015). NRF1 may function as a transcription factor that binds with the ARMC2 promoter and activates the expression of ARMC2, whereas ARMC2 may bind with PYCARD, a protein that functions as a key mediator in apoptosis and inflammation and mediates the assembly of large signaling complexes in the inflammatory and apoptotic signaling pathways via the activation of caspase, which leads to the processing and secretion of proinflammatory cytokines (STRING, 2021; Pathway Commons, 2019; Pierce et al., 2014; Mathelier et al., 2014). The function of PYCARD, as an activating adapter in different types of inflammasomes, is mediated by the pyrin and CARD domains and their homotypic interactions (Stehlik et al., 19502003; Manji et al., 2002; Srinivasula et al., 2002). The binding between ARMC2 and PYCARD, which involves the functional domain of pyrin and the CARD domain of PYCARD, may interfere with the activating effect of PYCARD. Consequently, ARMC2 may inhibit COPD progression and improve pulmonary function through the NRF1/ARMC2/PYCARD/caspase&NF-κB pathway.

#### 3.1.2. Primary Ciliary Dyskinesia (PCD)

According to previous studies, the ARMC3 gene may control human lung ciliogenesis and is coexpressed with the transcription factor FOXJ1, which activates CFAP157 expression (Lonergan et al., 2006; Wallmeier et al., 2019). ARMC4 is an axon protein that is needed for the correct positioning and anchoring of ODAs. A lack of ARMC4 leads to a decrease in the number of ODAs and causes severe ciliary dyskinesia and airway disease (Hjeij et al., 2013). CCDC151 encodes an axonemal coiled-coil protein, and its mutation blocks CCDC151 from entering respiratory tract cilia, resulting in failed assembly between the ODA component DNAH5 and the ODA-related components CCDC114 and ARMC4 in axons (Hjeij et al., 2014), causing ciliary dyskinesia.

ARMC9 is located in the cilia matrix and is upregulated during ciliogenesis. The middle section of the cilia contains microtubules (tubes A and B), and ARMC9 exerts a negative regulatory effect on the length of cilia B tubes (Louka et al., 2018). TOG array regulator of axonemal microtubules 1 (TOGARAM1) interacts with ARMC9. Therefore, a mutation in TOGARAM1 will destroy the interaction between TOGARAM1 and ARMC9, leading to short cilia and causing a decrease in axon acetylation and polyglutamyl (Latour et al., 2020).

### 3.2. Diseases of the Endocrine System

#### 3.2.1. Primary Aldosteronism (PA) and Primary Bilateral Macronodular Adrenal Hyperplasia (PBMAH)

ARMC5, which contains an N-terminal armadillo repeat domain and a C-terminal BTB domain, both of which are docking platforms for many proteins, is expressed in most endocrine tissues, including the pituitary gland, adrenal gland, and pancreas (Berthon et al., 2017b). On the one hand, ARMC5 inhibits PKA activation to restrain the Wnt/β-catenin pathway and thus inhibit Axins, Lef1, and Cyclin D1 expression (Berthon et al., 2017a), and on the other hand, although ARMC5 inhibits the cell cycle, the BTB domain of ARMC5 can interact with CUL3, which causes ARMC5 to be ubiquitinated and further degraded by proteases and thereby promotes the cell cycle (Cavalcante et al., 2020). Moreover, the missense mutation in the BTB domain of ARMC5 in more than 25–50% of patients with PBMAH, adrenal cortex tumors, and PA is not able to interact and be degraded by the CUL3/proteasome or to alter the cell cycle (Zilbermint et al., 2015; Berthon and Bertherat, 2020; Cavalcante et al., 2020), which leads to deregulation of cell cycle progression. In addition, ARMC5 mutations can cause increased PCNA expression, which is also a possible cause of these diseases (Conceição et al., 2020).

#### 3.2.2. Type 2 Diabetes (T2B)

ARMC1, which is correlated with mitochondrial-related diseases, is a subunit of the mammalian mitochondrial contact site and cristae organizing system/mitochondrial intermembrane space bridging complex (MICOS/MIB) (Wagner et al., 2019). ARMC1 binds to the outer mitochondrial membrane through an interaction between its armadillo repeat domain and solute carrier family 25 member 46 (SLC25A46) (STRING, 2021; Pathway Commons, 2019; Pierce et al., 2014) (Figures 3A, B). SLC25A46 promotes mitochondrial fission and prevents the formation of hyperfilamentous mitochondria (Abrams et al., 2015). Therefore, its overexpression may lead to fragmented mitochondria. Interestingly, mitochondria lacking the ARMC1 protein show no defects in cristae structure, respiration, or protein content but appear fragmented and have a reduced exercise capacity (Wagner et al., 2019). This finding suggests that the binding of ARMC1 to SLC25A46 leads to dysfunction in SLC25A46 and thus regulates mitochondrial fission and distribution. A growing number of studies have shown that mitochondrial dysfunction plays a key role in diabetes (Chow et al., 2017; Chan, 2020). Thus, ARMC1 may interact and interfere with SLC25A46, which leads to mitochondrial dysfunction and thus the promotion of diabetes.

When the glucose concentration increases, the N-terminus of ARMC9, which is similar to GID8, mediates the ubiquitin-proteasome-dependent catabolite degradation of fructose-1,6-bisphosphatase (FBP) (Regelmann et al., 2003), which suggests a hypoglycemic effect of ARMC9. Mutations in this domain may cause glucose metabolism disorders, such as diabetes.

ARMC10, which is located in mitochondria, interacts with the KIF5/Miro1-2/Trak2 complex to prevent Aβ-induced mitochondrial division (Serrat et al., 2014). In addition, the S45 site of ARMC10 can be phosphorylated by AMPK, which participates in the dynamic regulation of AMPK-mediated mitochondrial division and fusion (Chen et al., 2019). A growing number of studies have shown that mitochondrial dysfunction plays a key role in diabetes (Chow et al., 2017; Chan, 2020), which suggests that ARMC10 inhibits the progression of diabetes by interacting with the KIF5/Miro/Trak2 complex.

ARMCX3 interacts with the Kinesin/Miro/Trak2 complex in a calcium-dependent manner to regulate mitochondrial dynamics and transport (López-Doménech et al., 2012). The Wnt/PKC pathway also indirectly regulates the distribution and dynamics of mitochondria by degrading ARMCX3 (Serrat et al., 2013). Therefore, ARMCX3 may inhibit the progression of diabetes by interacting with the Kinesin/Miro/Trak2 complex.

### 3.3. Diseases of the Reproductive System

#### 3.3.1. Cryptorchidism (CC)

The partial deletion of ARMC4 may lead to male CC (Mroczkowski et al., 2014). This disease is characterized by deregulation of embryonic development or cell proliferation. The N-terminus of ARMC4, which is similar to GSK3B-interacting protein, may interact with GSK3B to help control the destabilizing phosphorylation of β-catenin and thus inhibit the Wnt/β-catenin pathway (Pierce et al., 2014). Because the Wnt/β-catenin pathway negatively regulates the embryonic mitotic cell cycle (Dema et al., 2016), we hypothesize that ARMC4 interacts with GSK3B, which leads to the destabilizing phosphorylation of β-catenin to inhibit the Wnt/β-catenin pathway and thus promote embryonic development.

#### 3.3.2. Infertility

The expression of ARMC3 is significantly increased in highly active sperm (D’Amours et al., 2019), whereas the deletion of exon 11 of the ARMC3 gene may alter the reading frame, leading to premature termination of translation, which causes sperm defects and thus male infertility (Pausch et al., 2016). Interestingly, ARMC3 exon 11 encodes amino acids 476–521 of ARMC3, which is located across the end of the last armadillo repeat sequence. Therefore, the deletion of exon 11 of ARMC3 leads to absence of the sequence after the last armadillo repeat in the C-terminal region, which is analogous to that in ribonuclease (Figures 1, 2). AKAP3 may have a subtle connection with ARMC3 (STRING, 2021; Pathway Commons, 2019). First, AKAP3 is an A-kinase anchor protein that functions as a regulator of both motility-associated and head-associated functions such as capacitation and the acrosome reaction via its interaction with cAMP-dependent protein kinase A (PKA) (Vijayaraghavan et al., 1999). Second, the ribonuclease-like domain on the C-terminus rather than the armadillo repeat domain may correlate with AKAP3. Third, the C-terminus of ARMC3, as a ribonuclease, is unlikely to interact with AKAP3 directly. Nevertheless, PKA activation is inhibited competitively by PKIA, which interacts with the catalytic subunit of the enzyme after the cAMP-induced dissociation of its regulatory chains (Vetter et al., 2011). This finding suggests that the C-terminus of ARMC3 hydrolyzes PKIA mRNA as a ribonuclease to rescue the competitive inhibition of PKA and thus promote interaction between AKAP3 and PKA to positively promote capacitation and the acrosome reaction.

It has also been reported that ARMCX4 promotes the differentiation of SSCs, and ARMCX4 mutations can cause male infertility (Lu et al., 2021). DPAGT1 catalyzes N-glycosylation (a posttranslational modification) (Dong et al., 2018). ARMCX4 interacts with DPAGT1, which suggests that ARMCX4 inhibits male infertility by interacting with DPAGT1 (STRING, 2021; Pathway Commons, 2019; Pierce et al., 2014).

### 3.4. Developmental and Congenital Diseases

#### 3.4.1. Fragile X Syndrome (FXS)

It has been reported that the absence of ARMCX1 enhances the regeneration of hematopoietic stem cells (Holmfeldt et al., 2016) and may thus regulate leukemia, myelodysplastic syndrome (MDS), metastatic cancer, and autoimmune diseases (AIDs) (Calvi and Link, 2015; Swart et al., 2017). EZH2 is a polycomb group (PcG) protein and a catalytic subunit of the PRC2/EED-EZH2 complex, which methylates “Lys-9” (H3K9me) and “Lys-27” (H3K27me) of histone H3 to result in transcriptional repression of the affected target gene (McCabe et al., 2012; Hübner et al., 2019). ARMCX1 may interact with EZH2, which leads to target gene methylation and thus inhibition of the regeneration of hematopoietic stem cells (STRING, 2021; Pathway Commons, 2019; Pierce et al., 2014). The same may also be true for ARMCX2 because the length of the whole sequence and the lengths of the two armadillo repeat domains of ARMCX1 and ARMCX2 are very similar. This finding explains why ARMCX2 is upregulated in patients with FXS (Rosales-Reynoso et al., 2010), which is characterized by methylation of the FMR1 promoter region (Bagni and Oostra, 2013), and how ARMCX2 participates in the development of a variety of tissues during embryogenesis, particularly testicular tissue differentiation (Smith et al., 2005).

#### 3.4.2. Glycosylation Disease (GD)

DPAGT1 catalyzes N-glycosylation (a posttranslational modification) (Dong et al., 2018). ARMCX4 interacts with DPAGT1, which suggests that ARMCX4 inhibits congenital myasthenic syndrome (CMS), GD, male infertility, and neonatal maxillofacial deformities by interacting with DPAGT1 (STRING, 2021; Pathway Commons, 2019; Pierce et al., 2014).

#### 3.4.3. Neonatal Maxillofacial Deformity (NMD)

The partial deletion of ARMC4 may lead to maxillofacial deformities in newborns (Okamoto et al., 2012). This disease is characterized by deregulation of embryonic development or cell proliferation. The N-terminus of ARMC4, which is similar to GSK3B-interacting protein, may interact with GSK3B to help control the destabilizing phosphorylation of β-catenin and thus inhibit the Wnt/β-catenin pathway (Pierce et al., 2014). Because the Wnt/β-catenin pathway regulates the embryonic mitotic cell cycle negatively and tumorigenesis positively (Dema et al., 2016), we hypothesize that ARMC4 interacts with GSK3B, and this interaction leads to the destabilizing phosphorylation of β-catenin to inhibit the Wnt/β-catenin pathway and thus promote embryonic development.

It has also been reported that ARMCX4 mutations can cause neonatal maxillofacial deformities (Okamoto et al., 2012). DPAGT1 catalyzes N-glycosylation (a posttranslational modification) (Dong et al., 2018). ARMCX4 interacts with DPAGT1, which suggests that ARMCX4 inhibits neonatal maxillofacial deformities by interacting with DPAGT1 (STRING, 2021; Pathway Commons, 2019; Pierce et al., 2014).

#### 3.4.4. Malformation (MF) and Osteogenesis Imperfecta (OI)

The N-terminus of ARMC9, which is similar to GID8, interacts with DCR2 and phosphatase CLN3 or SWI4 to regulate cell cycle progression (Pathak et al., 2004). The N-terminus of ARMC9, which is analogous to SRP1, interacts with multiple components of the cell nucleus that are needed for mitosis and regulates the normal function of microtubules and spindle pole bodies as well as nuclear integrity (Küssel and Frasch, 1995). Both domains on the N-terminus of ARMC9 may regulate the cell cycle, although through different pathways, which strongly suggests that ARMC9 is correlated with the cell cycle. This finding also suggests that a mutation or deletion in this area causes cell cycle stagnation, which leads to a congenital defect, MF, or OI.

#### 3.4.5. Waardenburg Syndrome (WS) Type II

Although ARMCX3 does not possess intrinsic transcriptional activity, it does enhance transactivation of the nicotinic acetylcholine receptor (nAChR) gene promoter via Sox10 and Wnt/β-catenin signaling in neuron-like cell lines and contributes to neural crest development and cell differentiation (Mou et al., 2009; Mirra et al., 2016). Sox10, whose mutations cause WS (Zhang et al., 2012), is a transcription factor that plays a central role in developing and mature glia, oligodendrocyte maturation, and central nervous system (CNS) myelination by activating the expression of target genes such as DUSP15 and MYRF (Zhang et al., 2012). Therefore, ARMCX3 may relieve the progression of WS type II by activating Sox10.

#### 3.4.6. Joubert Syndrome (JS)

ARMC9 is located in the cilia matrix and is upregulated during ciliogenesis. The middle section of the cilia contains microtubules (tubes A and B), and ARMC9 exerts a negative regulatory effect on the length of cilia B tubes (Louka et al., 2018). TOG array regulator of axonemal microtubules 1 (TOGARAM1) interacts with ARMC9. Therefore, a mutation in TOGARAM1 will destroy the interaction between TOGARAM1 and ARMC9, leading to short cilia and causing a decrease in axon acetylation and polyglutamyl chains (Latour et al., 2020). Variations in ARMC9 (stop gain, missense, splice site, and single exon deletion) can lead to JS, which causes hypotonia, ataxia, abnormal eye movements, neurodevelopmental defects, and various cognitive impairments (Van De Weghe et al., 2017).

### 3.5. Tumors

#### 3.5.1. Neuroblastoma

ARMC6 is the target gene of MYCN and indicates a poor prognosis for patients with neuroblastoma (NB) (Wang et al., 2020a). The transcription factor MYCN forms a heterodimer with the related transcription factor MAX and binds to the E-box DNA consensus sequence together to regulate the transcription of specific target genes (Beierle et al., 2007), including ARMC6 (Mathelier et al., 2014). Moreover, the additional domain on the N-terminus of ARMC6 is analogous to that of Inscuteable, which plays a major role in controlling asymmetric cell division and activating the NOTCH signaling pathway in neuroblasts (Udolph et al., 2009). The NOTCH signaling pathway regulates target gene expression through its hydrolysis fragment NICD or through ICN binding with the transcription factor CSL. This finding suggests that ARMC6 promotes NB tumorigenesis through the MYCN&MAX/ARMC6/NOTCH/NICD/CSL pathway.

Both ARMC12 and retinoblastoma binding protein 4 (RBBP4) are upregulated in NB tissues and are associated with a poor prognosis in patients (Li et al., 2018). It has been reported that ARMC12 interacts with RBBP4 and promotes the formation and activity of polycomb repressive complex 2, which leads to transcriptional inhibition of tumor suppressor genes (CADM1, EGLN3, HRK, HS6ST3, SMAD9) and thus the promotion of NB proliferation (Li et al., 2018).

#### 3.5.2. Glioma

ARMC1, which is correlated with mitochondrial-related diseases, is a subunit of the mammalian mitochondrial contact site and cristae organizing system/mitochondrial intermembrane space bridging complex (MICOS/MIB) (Wagner et al., 2019). ARMC1 binds to the outer mitochondrial membrane through an interaction between its armadillo repeat domain and solute carrier family 25 member 46 (SLC25A46) (STRING, 2021; Pathway Commons, 2019; Pierce et al., 2014) (Figures 3A,B). SLC25A46 promotes mitochondrial fission and prevents the formation of hyperfilamentous mitochondria (Abrams et al., 2015), and its overexpression may thus lead to fragmented mitochondria. Interestingly, mitochondria lacking the ARMC1 protein show no defects in cristae structure, respiration, or protein content but appear fragmented and have a reduced exercise capacity (Wagner et al., 2019). This finding suggests that the binding of ARMC1 to SLC25A46 leads to dysfunction in SLC25A46 and thus regulates mitochondrial fission and distribution. A growing number of studies have shown that mitochondrial dysfunction plays a key role in glioma (Jin et al., 2017; Chan, 2020). Thus, ARMC1 may interact and interfere with SLC25A46, which leads to mitochondrial dysfunction and thus promoting glioma tumorigenesis.

ARMC5 contains an N-terminal armadillo repeat domain and a C-terminal BTB domain, both of which are docking platforms for many proteins. On the one hand, ARMC5 inhibits PKA activation to restrain the Wnt/β-catenin pathway and thus inhibit Axins, Lef1, and Cyclin D1 expression (Berthon et al., 2017a), and on the other hand, although ARMC5 inhibits the cell cycle, the BTB domain in ARMC5 can interact with CUL3, which causes ARMC5 to be ubiquitinated and further degraded by proteases and thereby promotes the cell cycle (Cavalcante et al., 2020). Moreover, the missense mutation in the BTB domain of ARMC5 is not able to interact and be degraded by the CUL3/proteasome or alter the cell cycle (Zilbermint et al., 2015; Berthon and Bertherat, 2020; Cavalcante et al., 2020), which leading to deregulation of cell cycle progression. In addition, ARMC5 mutations can cause increased PCNA expression, which is also a possible cause of glioma (Conceição et al., 2020). Overall, ARMC5 plays a key role in regulating cell cycle progression. This finding is in accordance with the result that nuclear respiratory factor 1 (NRF1), a transcription factor, is highly active in astrocytoma and can indirectly regulate the cell cycle by targeting ARMC5 to promote glioma proliferation (Bhawe et al., 2020). The finding also illustrates why the knockout of ARMC5 inhibits T cell proliferation and differentiation into TH1 and TH17 cells and increases T cell apoptosis (Hu et al., 2017) and why ARMC5 mutations may cause meningioma (Elbelt et al., 2015).

ARMC10 may be a myeloid tumor suppressor gene (Curtiss et al., 2005). Moreover, temozolomide (TMZ)-resistant glioma cell lines are characterized by many activated functions, such as EMT, Wnt signal transduction, and the immune response, in which ARMC10 may play a key role (Cai et al., 2020). However, there is no additional domain other than a central armadillo repeat domain, which suggests that ARMC10 is related to myeloid tumors and gliomas through the interaction of its armadillo repeat domain with other proteins. Thus, ARMC10 may interact and interfere with EPHA1, TCEA2, and SMARCD1 through its armadillo repeat domain (STRING, 2021; Pathway Commons, 2019; Pierce et al., 2014). EPHA1 is a receptor tyrosine kinase that may promote the expression of vascular endothelial growth factor (VEGF) and matrix metalloproteinase (MMP)-2 and MMP-9 by regulating ILK and downstream RHOA and RAC and thus promote tumorigenesis and angiogenesis (Yamazaki et al., 2009; Chen et al., 2010). Both TCEA2 and SMARCD1 can bind to DNA, and this binding regulates template transcription to promote the Wnt/β-catenin pathway and thus promotes EMT and the immune response (Umehara et al., 1995; Otsuki et al., 2001; Sijbrandi et al., 2002; Hsiao et al., 2003; Kalluri and Neilson, 2003; Yook et al., 2006; Liu et al., 2013). This finding suggests that ARMC10 inhibits tumorigenesis and angiogenesis through the ARMC10/EPHA1/ILK/RHOA/ROCK/VEGF&MMP pathway and inhibits EMT and the immune response through the ARMC10/TCEA2&SMARCD1/Wnt/β-catenin pathway. Moreover, ARMC10 interacts with TMZ to relieve the effect of TMZ and thus induces TMZ drug-resistant glioma. ARMC10 has two potential ligand-binding pockets (Figure 3C). The O atoms of THR229 and HIS271 form hydrogen bonds with TMZ, whereas THR85, ASP86, ASP90, and ASN123 form noncovalent polar bonds with TMZ (Figures 3D,E). This finding suggests that TMZ, as a ligand, can bind to the ligand-binding pockets of ARMC10, and this binding results in deficiency in the concentration of TMZ in patients with glioma who undergo TMZ chemotherapy. A long duration of this state may cause TMZ drug resistance.

#### 3.5.3. Colorectal Cancer

It has been reported that ARMC4 is a tumor suppressor gene, and its mutation may cause colorectal cancer (Liang et al., 2019), which is characterized by cell proliferation. The N-terminus of ARMC4, which is similar to GSK3B-interacting protein, may interact with GSK3B to help control the destabilizing phosphorylation of β-catenin and thus inhibit the Wnt/β-catenin pathway (Pierce et al., 2014). Because the Wnt/β-catenin pathway regulates the embryonic mitotic cell cycle negatively and tumorigenesis positively (Dema et al., 2016), we hypothesize that ARMC4 interacts with GSK3B and that this interaction leads to the destabilizing phosphorylation of β-catenin to inhibit the Wnt/β-catenin pathway and thus promote embryonic development and inhibit tumorigenesis.

#### 3.5.4. Other Cancers

A growing number of studies related to ARMC8 are focusing on tumorigenesis, particularly tumor metastasis. On the one hand, in mammalian cells, ARMC8 is a key component of the C-terminal of lissencephaly type-1-like homology motif (CTLH) complex, which has E-3 ligase activity and is related to all basic biological processes and functions of the PI3 kinase, Wnt, TGF-β, and NF-κB pathways to regulate proliferation, survival, programmed cell death, cell adhesion, migration and other activities (Huffman et al., 2019). On the other hand, ARMC8 binds to the N-terminal sequence of α-catenin (amino acids 82–148) and promotes its degradation (Suzuki et al., 2008) to regulate cell adhesion. Almost all studies on the ARMC8 functions in promoting tumorigenesis and metastasis were based on these two mechanisms. For example, regarding cell adhesion and tumor metastasis, ARMC8 upregulates matrix metalloproteinase-7 and Snail and degrades α-catenin to disrupt the α-catenin/E-cadherin complex and promote the migration and invasion of hepatocellular carcinoma, breast cancer, lung adenocarcinoma, osteosarcoma, hepatocellular carcinoma, ovarian cancer, bladder cancer, colon cancer, and others (Jiang et al., 2015a; Jiang et al., 2015b; Zhao et al., 2016; Gul et al., 2019). Concerning E-3 ligase activity and other related biological processes, ARMC8 is involved in the regulation of pyrimidine metabolism, the pentose phosphate pathway, and the cellular functions of integrin signaling by controlling the proliferation and differentiation of lung adenocarcinoma (Amin et al., 2015); ARMC8 upregulates the expression of β-catenin, cyclin D1, MMP7, and c-Myc induced by TGF-β1 by activating the canonical Wnt signaling pathway and thereby promote the proliferation, migration, invasion and epithelial-mesenchymal transition (EMT) of lung cancer and bladder cancer cells (Xie et al., 2014; Jiang et al., 2016; Liang et al., 2017).

The N-terminus of ARMC9, which is similar to GID8, may interact with DCR2 and the phosphatase CLN3 or SWI4 to regulate cell cycle progression (Pathak et al., 2004). The N-terminus of ARMC9, which is analogous to SRP1, interacts with multiple components of the cell nucleus that are needed for mitosis and regulates the normal function of microtubules and spindle pole bodies as well as nuclear integrity (Küssel and Frasch, 1995). Both domains on the N-terminus of ARMC9 may regulate the cell cycle, although through different pathways, which strongly suggests that ARMC9 is correlated with the cell cycle. This finding also suggests that a mutation or deletion in this area causes cell cycle stagnation, which leads to cancers.

Because the cyclic AMP response element (CRE) and the E-box site are important *cis*-regulatory elements for ARMCX1 promoter activity and considering the same location of ARMCX1, ARMCX2, and ARMCX3 at Xq21.33-q22.2, the expression of ARMCX1, ARMCX2, and ARMCX3 is also regulated by CRE binding protein (CREB) and the Wnt/β-catenin signaling pathway (Iseki et al., 2010). The ARMCX1∼3 promoters are highly methylated in various tumors and thus inhibit their expression, which leads to rectal cancer, breast cancer, gastric cancer, and other cancers through the JAK1/STAT3, NF-κB, AKT/Slug, and PAR-1/ρGTPase signaling pathways (Iseki et al., 2012; Gao et al., 2015; Du et al., 2017; Pang et al., 2018; Zhu et al., 2019; Wang et al., 2020b). Interestingly, although the mRNA expression of ARMCX1 and ARMCX2 is significantly reduced in lung cancer, prostate cancer, colon cancer, pancreatic cancer, and ovarian cancer, these mRNAs are overexpressed in sarcoma, NB, cervical cancer, and glioma (Kurochkin et al., 2001; Zeng et al., 2015). ARMCX6 is also overexpressed in cervical cancer cells, the pancreas, and the spleen (Kusama et al., 2010). Regarding tissue development and differentiation, it has been reported that the absence of ARMCX1 enhances the regeneration of stem cells (Holmfeldt et al., 2016) and may thus regulate metastatic cancer (Calvi and Link, 2015; Swart et al., 2017). EZH2 is a polycomb group (PcG) protein and a catalytic subunit of the PRC2/EED-EZH2 complex, which methylates “Lys-9” (H3K9me) and “Lys-27” (H3K27me) of histone H3 and leads to transcriptional repression of the affected target gene (McCabe et al., 2012; Hübner et al., 2019). ARMCX1 may interact with EZH2, and this interaction leads to target gene methylation and thus inhibition of the regeneration of stem cells (STRING, 2021; Pathway Commons, 2019; Pierce et al., 2014). The same may also be true for ARMCX2 because the length of the whole sequence and the lengths of the two armadillo repeat domains of ARMCX1 and ARMCX2 are very similar. Moreover, although ARMCX3 does not possess intrinsic transcriptional activity, it does enhance transactivation of the nAChR gene promoter via Sox10 and Wnt/β-catenin signaling in neuron-like cell lines and contributes to neural crest development and cell differentiation (Mou et al., 2009; Mirra et al., 2016). A previous study suggested that the nAChR is related to cancers (Schaal et al., 2015); therefore, ARMCX3 may promote the progression of cancers. Concerning mitochondrial-related functions, ARMCX3 interacts with the Kinesin/Miro/Trak2 complex in a calcium-dependent manner to regulate neuronal mitochondrial dynamics and transport (López-Doménech et al., 2012). The Wnt/PKC pathway also indirectly regulates the distribution and dynamics of mitochondria by degrading ARMCX3 (Serrat et al., 2013). Therefore, ARMCX3 may inhibit the progression of cancers by interacting with the Kinesin/Miro/Trak2 complex.

### 3.6. Diseases of the Nervous System

#### 3.6.1. Alzheimer’s Disease (AD)

ARMC1 binds to the outer mitochondrial membrane through an interaction between its armadillo repeat domain and solute carrier family 25 member 46 (SLC25A46) (STRING, 2021; Pathway Commons, 2019; Pierce et al., 2014) (Figures 3A,B). SLC25A46 promotes mitochondrial fission and prevents the formation of hyperfilamentous mitochondria (Abrams et al., 2015). Therefore, its overexpression may lead to fragmented mitochondria. Interestingly, mitochondria lacking the ARMC1 protein show no defects in cristae structure, respiration, or protein content but appear fragmented and have a reduced exercise capacity (Wagner et al., 2019). This finding suggests that the binding of ARMC1 to SLC25A46 leads to dysfunction in SLC25A46 and thus regulates mitochondrial fission and distribution. A growing number of studies have shown that mitochondrial dysfunction plays a key role in AD (Chow et al., 2017; Macdonald et al., 2018; Chan, 2020). Thus, ARMC1 may interact and interfere with SLC25A46, which leads to mitochondrial dysfunction and thus the promotion of AD.

The N-terminus of ARMC4, which is similar to GSK3B-interacting protein, interacts with GSK3B and recruits Drp1 to form a working complex, which promotes the phosphorylation of Drp1 and thus regulates mitochondrial elongation and neurite outgrowth (Loh et al., 2015). Moreover, the N-terminus of ARMC4 may interact with PKA to help control the stabilizing phosphorylation of β-catenin and thus promote the Wnt/β-catenin pathway, which regulates neuronal development (Chou et al., 2006; Dema et al., 2016). This finding suggests that ARMC4 promotes neuronal development and thus AD through the ARMC4/GSK3B/Drp1 or ARMC4/PKA/Wnt/β-catenin pathway.

The ARMC7 gene may be related to AD (Soleimani Zakeri et al., 2020). However, no additional domain is found on the terminals, and its sequence is almost entirely occupied by the armadillo repeat domain. Therefore, the relationship of ARMC7 to AD is mediated by its interaction with other proteins, such as APP(STRING, 2021; Pathway Commons, 2019; Pierce et al., 2014). APP is an amyloid-beta precursor protein that induces the clusterin/p53/Dkk1/Wnt/PCP/JNK pathway, which drives the upregulation of several genes, including EGR1, NAB2, and KLF10, to mediate neurotoxicity and tau phosphorylation and thus promotes the development of neuropathologies observed in AD (Killick et al., 2014). This finding suggests that ARMC7 interacts with APP to promote AD progression through the ARMC7/APP/clusterin/p53/Dkk1/Wnt/PCP/JNK pathway.

ARMC10, which is located in mitochondria and is highly expressed in the brain, interacts with the KIF5/Miro1-2/Trak2 complex to prevent Aβ-induced mitochondrial division and neuronal death (Serrat et al., 2014). In addition, the S45 site of ARMC10 can be phosphorylated by AMPK, which participates in the dynamic regulation of AMPK-mediated mitochondrial division and fusion (Chen et al., 2019). A growing number of studies have shown that mitochondrial dysfunction plays a key role in AD (Chow et al., 2017; Jin et al., 2017; Macdonald et al., 2018; Chan, 2020). Moreover, Aβ is correlated with AD (Killick et al., 2014; Weber et al., 2018), which suggests that ARMC10 inhibits the progression of AD by interacting with the KIF5/Miro/Trak2 complex.

Although ARMCX3 does not possess intrinsic transcriptional activity, it does enhance transactivation of the nAChR gene promoter via Sox10 and Wnt/β-catenin signaling in neuron-like cell lines and contributes to neural crest development and cell differentiation (Mou et al., 2009; Mirra et al., 2016). A growing number of studies have suggested that the nAChR is related to nicotine addiction, cognition, depression, hyperactivity disorder, cancer, and AD (Ma and Qian, 2019); therefore, ARMCX3 may promote the progression of AD. In addition, ARMCX3 interacts with the Kinesin/Miro/Trak2 complex in a calcium-dependent manner to regulate neuronal mitochondrial dynamics and transport (López-Doménech et al., 2012). The Wnt/PKC pathway also indirectly regulates the distribution and dynamics of mitochondria by degrading ARMCX3 (Serrat et al., 2013). Therefore, ARMCX3 may inhibit the progression of AD by interacting with the Kinesin/Miro/Trak2 complex.

#### 3.6.2. Parkinson’s Disease (PD)

ARMC1 binds to the outer mitochondrial membrane through an interaction between its armadillo repeat domain and solute carrier family 25 member 46 (SLC25A46) (STRING, 2021; Pathway Commons, 2019; Pierce et al., 2014) (Figures 3A,B). SLC25A46 promotes mitochondrial fission and prevents the formation of hyperfilamentous mitochondria (Abrams et al., 2015), and its overexpression may thus lead to fragmented mitochondria. Interestingly, mitochondria lacking the ARMC1 protein show no defects in cristae structure, respiration, or protein content but appear fragmented and have a reduced exercise capacity (Wagner et al., 2019). This finding suggests that the binding of ARMC1 to SLC25A46 leads to dysfunction in SLC25A46 and thus regulates mitochondrial fission and distribution. A growing number of studies have shown that mitochondrial dysfunction plays a key role in PD (Chow et al., 2017; Macdonald et al., 2018; Chan, 2020). Thus, ARMC1 may interact and interfere with SLC25A46, which leads to mitochondrial dysfunction and thus the promotion of PD.

The N-terminus of ARMC4, which is similar to GSK3B-interacting protein, interacts with GSK3B and recruits Drp1 to form a working complex, which promotes the phosphorylation of Drp1 and thus the regulation of mitochondrial elongation and neurite outgrowth (Loh et al., 2015). Moreover, the N-terminus of ARMC4 may interact with PKA to help control the stabilizing phosphorylation of β-catenin and thus promote the Wnt/β-catenin pathway, which regulates neuronal development (Chou et al., 2006; Dema et al., 2016). This finding suggests that ARMC4 promotes neuronal development and thus PD through the ARMC4/GSK3B/Drp1 or ARMC4/PKA/Wnt/β-catenin pathway.

ARMC10, which is located in mitochondria and is highly expressed in the brain, interacts with the KIF5/Miro1-2/Trak2 complex to prevent Aβ-induced mitochondrial division and neuronal death (Serrat et al., 2014). In addition, the S45 site of ARMC10 can be phosphorylated by AMPK, which participates in the dynamic regulation of AMPK-mediated mitochondrial division and fusion (Chen et al., 2019). A growing number of studies have shown that mitochondrial dysfunction plays a key role in PD (Macdonald et al., 2018; Chan, 2020). This finding suggests that ARMC10 inhibits the progression of PD by interacting with the KIF5/Miro/Trak2 complex. With respect to mitochondrial-related diseases, ARMCX3 interacts with the Kinesin/Miro/Trak2 complex in a calcium-dependent manner to regulate neuronal mitochondrial dynamics and transport (López-Doménech et al., 2012). The Wnt/PKC pathway also indirectly regulates the distribution and dynamics of mitochondria by degrading ARMCX3 (Serrat et al., 2013). Therefore, ARMCX3 may inhibit the progression of PD by interacting with the Kinesin/Miro/Trak2 complex. In addition, ARMCX3 is related to the regulation of cholesterol, fatty acids, glucose metabolism, and muscle development or hypertrophy because ARMCX3 methylation causes the downregulation of HDAC7 and FYN, which lead to a low birth weight (LBW), insulin resistance, and T2D (Broholm et al., 2020).

#### 3.6.3. Amyotrophic Lateral Sclerosis (ALS)

ARMC4 variants may cause ALS (Goldstein et al., 2019). The N-terminus of ARMC4, which is similar to GSK3B-interacting protein, interacts with GSK3B and recruits Drp1 to form a working complex, which promotes the phosphorylation of Drp1 and thus the regulation of mitochondrial elongation and neurite outgrowth (Loh et al., 2015). Moreover, the N-terminus of ARMC4 may interact with PKA to help control the stabilizing phosphorylation of β-catenin and thus promote the Wnt/β-catenin pathway, which regulates neuronal development (Chou et al., 2006; Dema et al., 2016). This finding suggests that ARMC4 promotes neuronal development and thus inhibits ALS through the ARMC4/GSK3B/Drp1 or ARMC4/PKA/Wnt/β-catenin pathway.

#### 3.6.4. Cerebral Amyloid Angiopathy (CAA)

The terminals of ARMC7 contain no additional domain, and its sequence is almost entirely occupied by the armadillo repeat domain. Therefore, the relationship of ARMC7 to diseases is mediated by its interaction with other proteins, such as APP (STRING, 2021; Pathway Commons, 2019; Pierce et al., 2014). APP is an amyloid-beta precursor protein that induces the clusterin/p53/Dkk1/Wnt/PCP/JNK pathway, which drives the upregulation of several genes, including EGR1, NAB2, and KLF10, to mediate neurotoxicity and tau phosphorylation (Killick et al., 2014). This finding suggests that ARMC7 interacts with APP to mediate neurotoxicity and tau phosphorylation through the ARMC7/APP/clusterin/p53/Dkk1/Wnt/PCP/JNK pathway. Because CAA is characterized by the pathologic deposition of amyloid-beta within cortical and leptomeningeal arteries, arterioles, and capillaries (Weber et al., 2018), we hypothesize that ARMC7 promotes CAA via its interaction with APP.

ARMC10, which is located in mitochondria and is highly expressed in the brain, interacts with the KIF5/Miro1-2/Trak2 complex to prevent Aβ-induced mitochondrial division and neuronal death (Serrat et al., 2014). Aβ is correlated with CAA (Killick et al., 2014; Weber et al., 2018). This finding suggests that ARMC10 inhibits the progression of CAA by interacting with the KIF5/Miro/Trak2 complex.

#### 3.6.5. Congenital Myasthenic Syndrome (CMS)

It has also been reported that ARMCX4 promotes the differentiation of stem cells (SCs), and ARMCX4 mutations can cause congenital diseases (Okamoto et al., 2012; Lu et al., 2021). DPAGT1 catalyzes N-glycosylation (a posttranslational modification) (Dong et al., 2018). ARMCX4 interacts with DPAGT1, which suggests that ARMCX4 inhibits CMS by interacting with DPAGT1 (STRING, 2021; Pathway Commons, 2019; Pierce et al., 2014).

#### 3.6.6. Nicotine Addiction, Cognition, Depression, and Hyperactivity Disorder

Although ARMCX3 does not possess intrinsic transcriptional activity, it does enhance transactivation of the nAChR gene promoter via Sox10 and Wnt/β-catenin signaling in neuron-like cell lines and contributes to neural crest development and cell differentiation (Mou et al., 2009; Mirra et al., 2016). A growing number of studies have suggested that the nAChR is related to nicotine addiction, cognition, depression, hyperactivity disorder, cancer, and AD (Sarter et al., 2009; Schaal et al., 2015; Laikowski et al., 2019; Ma and Qian, 2019); therefore, ARMCX3 may promote the progression of these diseases.

#### 3.6.7. Guillain-Barre Syndrome (GBS)

ARMCX3 enhances transactivation of the nAChR gene promoter via Sox10 and Wnt/β-catenin signaling in neuron-like cell lines and contributes to neural crest development and cell differentiation (Mou et al., 2009; Mirra et al., 2016). Moreover, Sox10 is a transcription factor that plays a central role in developing and mature glia, oligodendrocyte maturation, and central nervous system (CNS) myelination by activating the expression of target genes such as DUSP15 and MYRF(Zhang et al., 2012). Therefore, ARMCX3 may relieve the progression of GBS by activating Sox10/DUSP15 and MYRF.

### 3.7. Diseases of the Immune System

#### 3.7.1. Autoimmune Disease (AID)

It has been reported that the absence of ARMCX1 enhances the regeneration of hematopoietic stem cells (Holmfeldt et al., 2016) and may thus regulate AIDs (Swart et al., 2017). EZH2 is a polycomb group (PcG) protein and a catalytic subunit of the PRC2/EED-EZH2 complex, which methylates “Lys-9” (H3K9me) and “Lys-27” (H3K27me) of histone H3 and thereby leads to transcriptional repression of the affected target gene (McCabe et al., 2012; Hübner et al., 2019). ARMCX1 may interact with EZH2, and this interaction leads to target gene methylation and thus inhibition of the regeneration of hematopoietic stem cells (STRING, 2021; Pathway Commons, 2019; Pierce et al., 2014).

### 3.8. Diseases of the Circulatory System

#### 3.8.1. Hypertension

The ARMC3 gene may be related to spontaneous hypertension (Kinoshita et al., 2011). CRACR2A, a Ca2+-binding protein that plays a key role in store-operated Ca2+ entry (SOCE) by regulating CRAC channel activation, may interact with ARMC3 (STRING, 2021; Pathway Commons, 2019; Pierce et al., 2014). On the one hand, CRACR2A acts as a cytoplasmic calcium sensor that facilitates the clustering of ORAI1 and STIM1 at the junctional regions between the plasma membrane and the endoplasmic reticulum at low Ca2+ concentrations (Srikanth et al., 2010). On the other hand, hypertension is positively correlated with SOCE (Bhullar et al., 2019). This finding suggests that ARMC3 interacts and interferes with CRACR2A and thus inhibits the expression of ORAI1 and STIM1 to suppress hypertension.

### 3.9. Diseases of the Blood System

#### 3.9.1. Myelodysplastic Syndrome (MDS) and Leukemia

Regarding tissue development and differentiation, it has been reported that the absence of ARMCX1 enhances the regeneration of hematopoietic stem cells (Holmfeldt et al., 2016) and may thus regulate leukemia and MDS (Calvi and Link, 2015; Swart et al., 2017). EZH2 is a PcG protein and a catalytic subunit of the PRC2/EED-EZH2 complex, which methylates “Lys-9” (H3K9me) and “Lys-27” (H3K27me) of histone H3 and results in transcriptional repression of the affected target gene (McCabe et al., 2012; Hübner et al., 2019). ARMCX1 may interact with EZH2, and this interaction leads to target gene methylation and thus inhibits the regeneration of hematopoietic stem cells (STRING, 2021; Pathway Commons, 2019; Pierce et al., 2014).

## 4. Clinical Perspective

ACVMPs are correlated with many diseases, such as nervous system diseases, respiratory system diseases, circulatory system diseases, hematological system diseases, immune system diseases, reproductive system diseases, endocrine system diseases, and many types of tumors. Their biological function in one disease may be compatible with that in other diseases due to their similar structures, which indicates that a similar biological function or pathway exists in many diseases with an unclear mechanism. For example, both ARMC10 and ARMCX3 interact with the Miro/Trak2 complex in mitochondria and thus regulate mitochondrial function to inhibit the progression of AD, PD, diabetes, cancers, mitochondrial diseases, immune-related diseases, and other mitochondrial-related diseases. Because many ARMCs are correlated with mitochondria, an increasing number of ARMCs may regulate mitochondrial functions and related diseases. Moreover, ACVMPs may exert positive and negative effects depending on the disease and thus contribute to the dynamic balance. For example, ARMC1 may interact and interfere with SLC25A46 and thus lead to mitochondrial dysfunction, whereas ARMC10 may interact and activate the Miro/track complex to positively regulate mitochondrial function. This finding indicates the opposing functions of ARMCs and helps determine new biological functions to illustrate the related diseases. ACVMPs may also contribute to improvements in targeted therapy. For example, ARMC10 harbors potential ligand-binding pockets that may interact with TMZ, which leads to TMZ drug-resistant glioma. Therefore, mutations in or competitive inhibition of the domain may affect the chemical therapy of glioma. Overall, ACVMPs have many applications in clinical medicine.

## 5. Concluding Remarks

**FIGURE 4:**
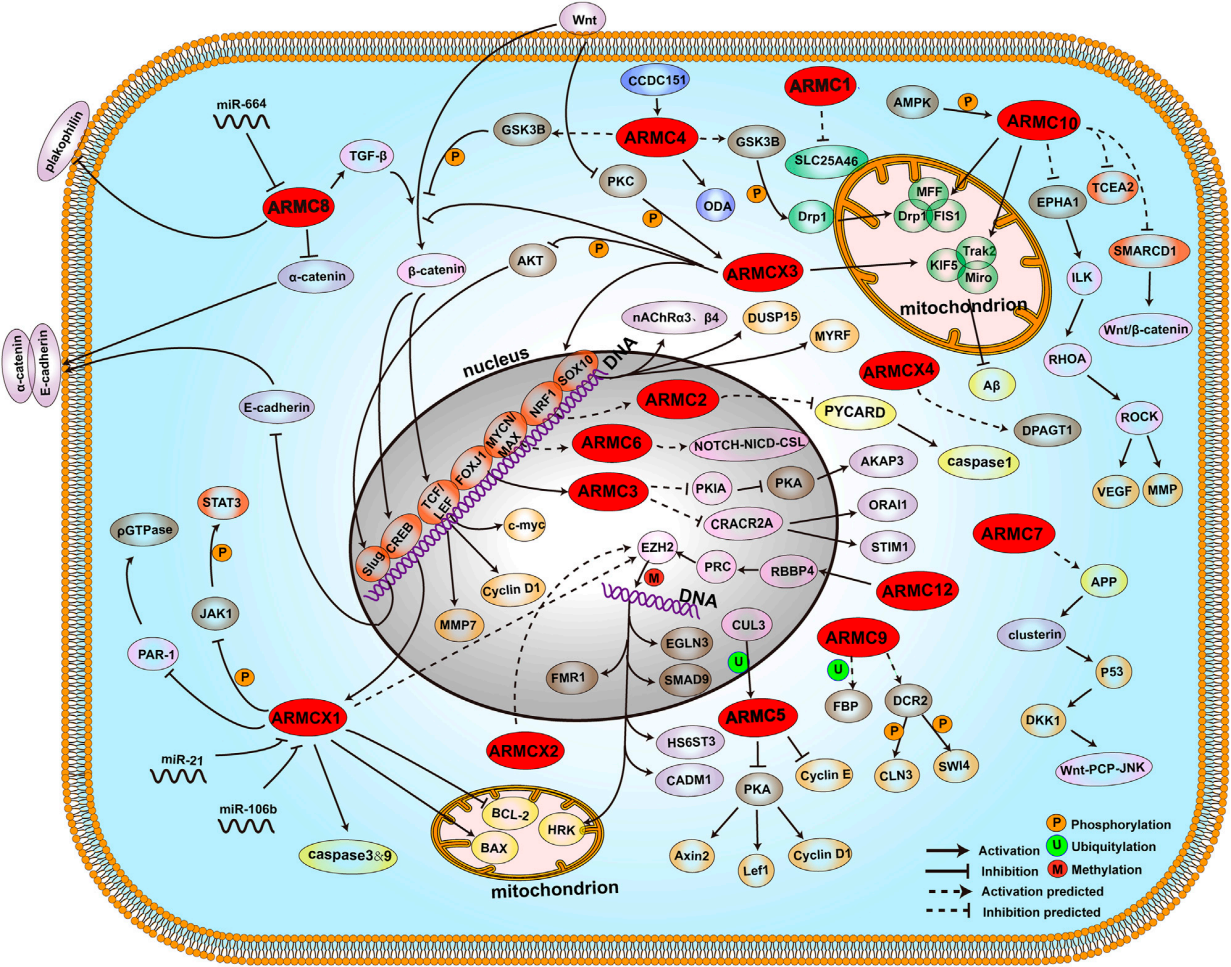
ARMCs and pathways. The locations of proteins and compounds are roughly depicted. Shown are the ARMC subfamily (red), transcription factors (orange), mitochondrial-related proteins (green), proliferation and cell cycle-related proteins (wheat), apoptosis-related proteins (yellow), modification-related proteins (sand), ODA-related proteins (blue), cadherin-related proteins (purple), and other proteins (pink). The phosphorylation modification, ubiquitylation modification, and methylation modification are shown as orange circles (P), green circles (U), and red circles (M), respectively.

ARMCs are widely expressed in eukaryotes. These proteins contain a similar domain that consists of tandem repeats with a length of approximately 42 amino acids, which constitutes the platform for protein-protein binding, and perform similar functions, such as the degradation or stabilization of target proteins. ACVMPs play a key role in many human diseases. Some of these proteins are unique to mammals and may have special functions. Here, we reviewed the structures of proteins in the ARMC subfamily with many unique domains that armadillo repeat-containing proteins usually do not have and then reviewed their biological functions and related diseases, including respiratory system diseases, endocrine system diseases, reproductive system diseases, congenital diseases, tumors, and nervous system diseases based on their structures. The review involves more than 30 diseases and 40 bypasses, including interactions and relationships between more than 100 proteins and signaling molecules (Table 1 and Figure 4). We recommend further classification of ARMC proteins into two categories: canonical ARMC proteins (e.g., β-catenin), which contain a central armadillo repeat, and noncanonical ARMC proteins, which contain N-terminal or C-terminal special domains in addition to the central armadillo repeat (e.g., ARMC1). Our research sheds light on these proteins through their structures, biological functions, and related diseases and provides a clearer understanding of the ARMC subfamily to facilitate more in-depth research and improve the treatment of related diseases.
